# Measurement of phospholipid lateral diffusion at high pressure by in situ magic-angle spinning NMR spectroscopy

**DOI:** 10.1038/s42004-025-01449-7

**Published:** 2025-02-14

**Authors:** Thomas M. Osborn Popp, Mithun Karthikeyan, Elias M. Herman, Andrew C. Dufur, Costantino Vetriani, Andrew J. Nieuwkoop

**Affiliations:** 1 Department of Chemistry and Chemical Biology, Rutgers University, Piscataway, NJ 08854 USA; 2 Department of Chemistry, Oregon State University, Corvallis, OR 97331 USA; 3 Department of Marine and Coastal Sciences, Rutgers University, New Brunswick, NJ 08901 USA; 4 Department of Biochemistry and Microbiology, Rutgers University, New Brunswick, NJ 08901 USA; 5 Present Address: Department of Chemistry, Oregon State University, Corvallis, OR 97331 USA

**Keywords:** Biophysical chemistry, Solid-state NMR, Membrane lipids, Phospholipids

## Abstract

The development of experimental methodologies that enable investigations of biochemistry at high pressure promises to yield significant advances in our understanding of life on Earth and its origins. Here, we introduce a method for studying lipid membranes at thermodynamic conditions relevant for life at deep sea hydrothermal vents. Using in situ high pressure magic-angle spinning solid state nuclear magnetic resonance spectroscopy (NMR), we measure changes in the fluidity of model microbial membranes at pressures up to 28 MPa. We find that the fluid-phase lateral diffusion of phospholipids at high pressure is significantly affected by the stoichiometric ratio of lipids in the membrane. Our results were facilitated by an accessible pressurization strategy that we have developed to enable routine preparation of solid state NMR rotors to pressures of 30 MPa or greater.

## Abstract (web summary)

Despite the significance of high pressure in the story of life on Earth, our current understanding of biochemistry at high pressure is limited, primarily due to technological challenges associated with using pressure as an experimental variable. Here, the authors report in situ high-pressure magic-angle spinning solid-state nuclear magnetic resonance spectroscopy to study the fluidity of model microbial membranes at pressures up to 28 MPa, showing a stoichiometric effect of lipids in the membrane on the phospholipid lateral diffusion at high pressure.

## Introduction

The discovery of life at deep sea hydrothermal vents flourishing at high pressures and temperatures has had considerable implications for our understanding of biology and the origins of life on Earth^1–3^. Hydrothermal vent ecosystems can be sustained entirely decoupled from the energy of the sun by thermopiezophilic anaerobic microorganisms that participate in chemosynthesis, the conversion of inorganic carbon into organic matter by the oxidation of inorganic molecules^4–8^. Given that thermopiezophiles have also been discovered within the deep subsurface of Earth, combined with the vastness of this putative microbial habitat, it is likely that a large portion of all biomass on earth is thermopiezophilic^9–14^. It has even been proposed that life on earth may have begun in the deep biosphere, and that such an environment could be a strong candidate for the existence of life on other planets^2,3,15^.

Despite the significance of high pressure in the story of life on Earth, our current understanding of biochemistry at high pressure is limited, primarily due to technological challenges associated with using pressure as an experimental variable. The highest pressure at which life is known to persist does not exceed 150 MPa, and while these pressures are not enough to impact covalent bonding, they can influence molecular conformations and intermolecular distances^3,16^. This is particularly important for phospholipid membranes, which must remain fluid in order to function properly^17^.

Previous experimental and theoretical investigations of phospholipid membranes subjected to high pressures have revealed that pressure acts to increase the packing and ordering of lipids within the membrane, resulting in an increase in the temperature required to transition between the solid-like gel state and the liquid-crystalline fluid phase^18–20^. These studies have also shown that the composition of the lipid mixture comprising the membrane can play a major role in modulating this transition temperature, suggesting structure-property roles for different lipid headgroups and acyl tails relating to membrane dynamics.

While the gel-fluid transition temperature is an important experimental proxy for membrane molecular dynamics, membranes are only functional for biological processes in the fluid state above the transition temperature^17^. In fact, it has been known that cells tightly regulate membrane fluidity by altering their composition of membrane lipids in response to changes in environmental conditions, as if to maintain a fluidity “set point,” in a phenomenon referred to as the homeoviscous response^21,22^. Though homeoviscous adaptation to temperature is well-documented across both eukaryotes and prokaryotes, less is known about the homeoviscous response to high pressure. Marine microorganisms that live at colder temperatures and high pressures have been observed to increase the ratio of unsaturated fatty acid tails to saturated fatty acids when grown under high pressure conditions, and some recent evidence suggests that some thermopiezophiles may respond to pressure by increasing the proportion of lipids with branched-chain fatty acid tails^23,24^. More comprehensive lipidomics studies involving the analysis of whole lipid molecules in pressure-adapted species can help to establish trends in the functional role of particular lipids in maintaining membrane fluidity at high pressure, with a recent hypothesis suggesting that organisms modulate the proportion of lipids that contribute negative curvature to the membrane in response to changes in pressure^25^.

Insight into the structure-property roles for lipids in their ability to modulate membrane dynamics in response to pressure requires methods for directly measuring membrane fluidity as a function of lipid composition. A major contributing factor to the fluidity of a membrane is the lateral self-diffusion of lipids within the plane of the membrane bilayer, which can be quantified by the lateral diffusion coefficient, *D*_lat_. There exist several methods for observing the lateral diffusion of molecules within membranes, including but not limited to fluorescence recovery after photobleaching (FRAP), fluorescence correlation spectroscopy (FCS), neutron scattering, electron paramagnetic resonance (EPR), and nuclear magnetic resonance (NMR)^21,26–30^. Recently, Macdonald and coworkers have developed a ^31^P magic-angle spinning (MAS) solid state NMR methodology based on the centerband-only detection of exchange (CODEX) pulse sequence that enables direct measurement of phospholipid *D*_lat_ in membrane vesicles^31–35^. The method has the advantage of requiring no tags or labeling schemes that could potentially interfere with lipid diffusion, and as it is based on detection of ^31^P NMR signal, the lateral diffusion of each phospholipid within a mixed-lipid membrane may be measured simultaneously, provided peaks can be spectrally resolved from one another.

In the present study, we extend the ^31^P CODEX NMR method to measure the lateral diffusion of phospholipid membranes using MAS sample rotors rated to contain pressures in situ up to 40 MPa at room temperature and up to 30 MPa at 250 °C^36,37^. Our NMR data were obtained at 55 °C at both ambient pressure and at 28 MPa on membranes composed of two phospholipids with identical tail compositions but differing headgroups: 1-palmitoyl-2-oleoyl-sn-glycero-3-phosphoethanolamine (16:0,18:1-PE, POPE), and 1-palmitoyl-2-oleoyl-sn-glycero-3-phosphoglycerol (16:0,18:1-PG, POPG). Our results show that the stoichiometric ratio of POPE to POPG in the membrane influences the extent to which phospholipid lateral diffusion is affected by high pressure. Beyond our experimental findings, we introduce a straightforward and cost-effective inert gas pressurization strategy that makes use of a modified three-stage manual pump, enabling easy access to pressures as high as 30 MPa or greater. Additionally, we provide a Python-based simulation and fitting tool with a graphical user interface for extracting phospholipid lateral diffusion coefficients from CODEX decay data. The approach we present here delivers unprecedented quantitative, lipid-specific experimental measurements of lateral diffusion under high pressure conditions and serves as an accessible platform for future investigations of high pressure membrane biophysics.

## Results and Discussion

### Strategy for routine and cost-effective preparation of samples at high pressure

**Fig. 1:**
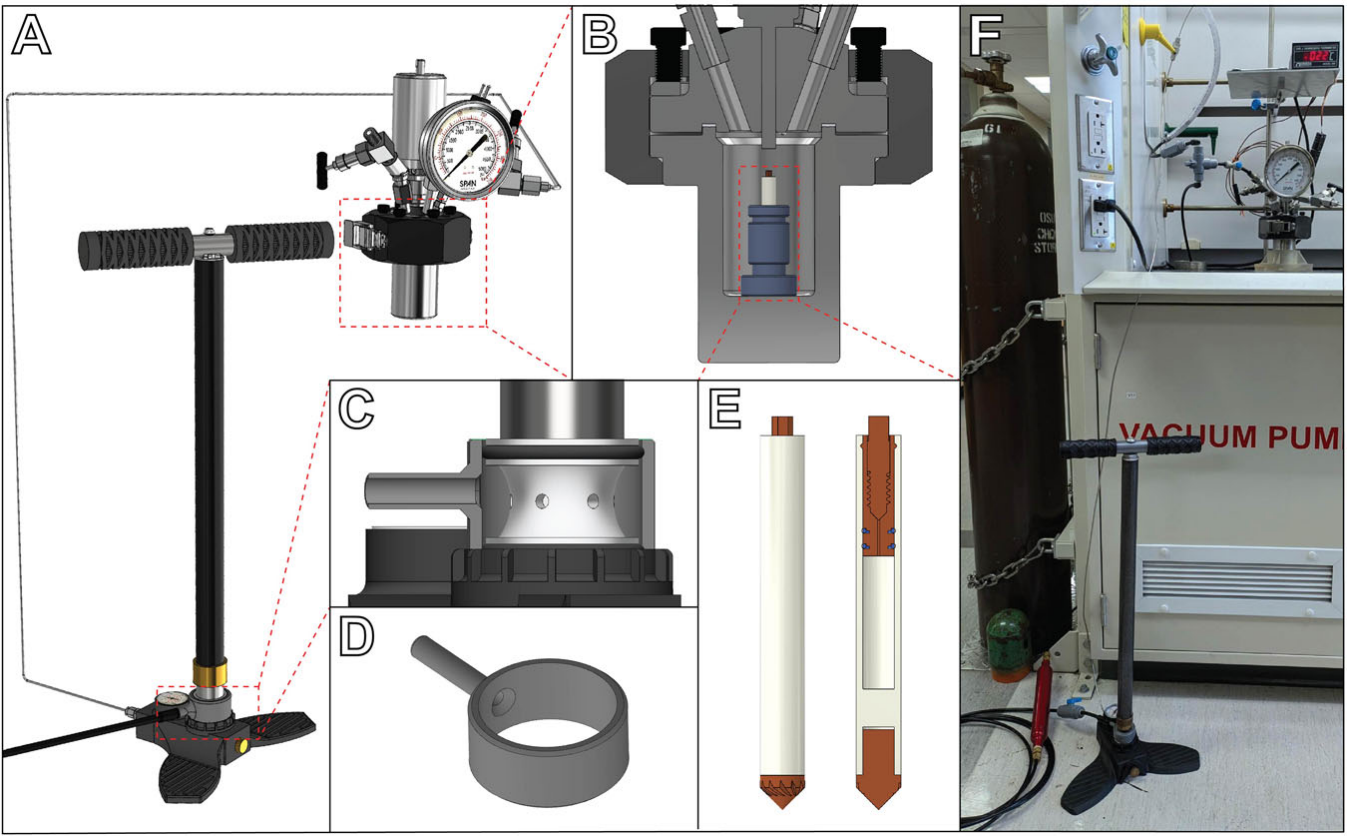
Apparatus for preparing high pressure MAS rotors. **A** Model of the high pressure apparatus used in this study. Inert gas at near-atmospheric pressure is fed into the base of a manual high pressure pump, which can then pressurize a 50 mL high pressure vessel up to 30–35 MPa. **B** Cross-section view of the high pressure vessel. A high pressure rotor is placed in the vessel held upright by a collet. **C** Cross section view of the gas intake of the manual pump and 3D-printable adapter. **D** 3D-printable adapter for the manual pump. **E** View with cross section of the 5 mm high pressure MAS rotor. The cap assembly acts as a one-way check valve, enabling pressurization of the rotor in the vessel even while sealed. **F** Photograph of the high pressure apparatus in the lab.

To prepare samples for in situ high pressure MAS NMR, we developed an apparatus that enables safe and routine access to pressures as high as 30 MPa or greater (Fig. 1). The apparatus uses inert gas as the pressurization medium, which is fed at near atmospheric pressure into the inlet of a high pressure pump to pressurize the contents of a 50 mL stainless steel vessel. As compressed gas cylinders typically contain only 13–18 MPa and are regulated down to lower pressures for routine use, safely applying pressures ranging from atmospheric up to values exceeding the original cylinder pressure requires some type of high pressure pump. While a high pressure syringe pump or gas booster pump might be the usual choice for this task, we have found a low-cost (~$250 USD) and effective alternative in the form of a manual pump originally designed for pressurizing air rifles. Although it resembles a low-pressure bicycle tire pump, the internal three-stage piston mechanism of this style of pump can pressurize a small volume from 0.1 to 30–35 MPa within minutes (at the cost of some physical exertion). Compared to commercially available high-pressure pumps—often quoted between $10,000-$50,000—this manual pump can offer at least a 100-fold reduction in cost. A benefit of this manual pumping strategy is a reduced likelihood of accidental overpressurization, as the physical work required to reach higher pressures results in clear and continuous feedback between the operator and the apparatus.

We modified this manual pump to accept an inert gas feed by installing a custom 3D-printed adapter on the o-ring seal of the pump intake (Fig. 1C, D). Helium was chosen as the pressure-transmitting medium, as its low solubility in both water and lipids results in a close approximation to hydrostatic pressure alone, while other inert gases like argon and even nitrogen can readily partition into membranes and result in significant changes in the pressure dependence of the gel-fluid transition temperature^38,39^. The top cap assembly of the high pressure MAS rotors used in our experiments acts as a one-way check valve, allowing the sealed rotor and its contents to be pressurized with helium when exposed to high pressure within the vessel, but not release that pressure when returned to ambient conditions^36^. We expect that our manual pump pressurization strategy can readily be applied in other contexts where pressure plays a key role, such as in investigations of high pressure chemical reactions, as manual pressurization with inert gas provides a relatively straightforward and accessible way to utilize pressure as an experimental variable.

### In situ variable temperature, high pressure MAS NMR of model membranes

The model membrane samples studied here represent simplified analogues of bacterial membranes, which typically contain PE and PG phospholipids as major components^40^. We prepared vesicle samples comprised of two different ratios of POPE and POPG: a POPG-rich sample, 3:1 POPE:POPG, and a POPG-dilute sample, 7:1 POPE:POPG, both of which fall within the physiologically relevant range of PE and PG concentrations observed in bacterial membranes. To enhance the biological significance of our results, we designed our investigation to examine these samples under conditions relevant for bacteria that thrive at deep-sea hydrothermal vents. While vent fluids can reach extreme temperatures exceeding 100 °C, most organisms in these ecosystems live in the immediate surroundings of the vents at temperatures between those of the vent fluids and the cold waters of the deep ocean. For this study, we selected our experimental conditions to mimic the optimal growth conditions of a piezophilic Campylobacterium species, *Nautilia sp*. strain PV-1, which was recently isolated from a hydrothermal vent on the East Pacific Rise 9° N and successfully grown in the lab at 55 °C and pH 5.5 at both low pressure (0.2 MPa) and high pressure (>20 MPa)^8^. It is worth noting that while the conditions chosen for our NMR experiments are at a moderate temperature, the MAS rotors employed here are capable of supporting experiments up to 250 °C and 30 MPa, enabling investigations across a much greater range of conditions relevant for life at deep sea hydrothermal vents.

**Fig. 2:**
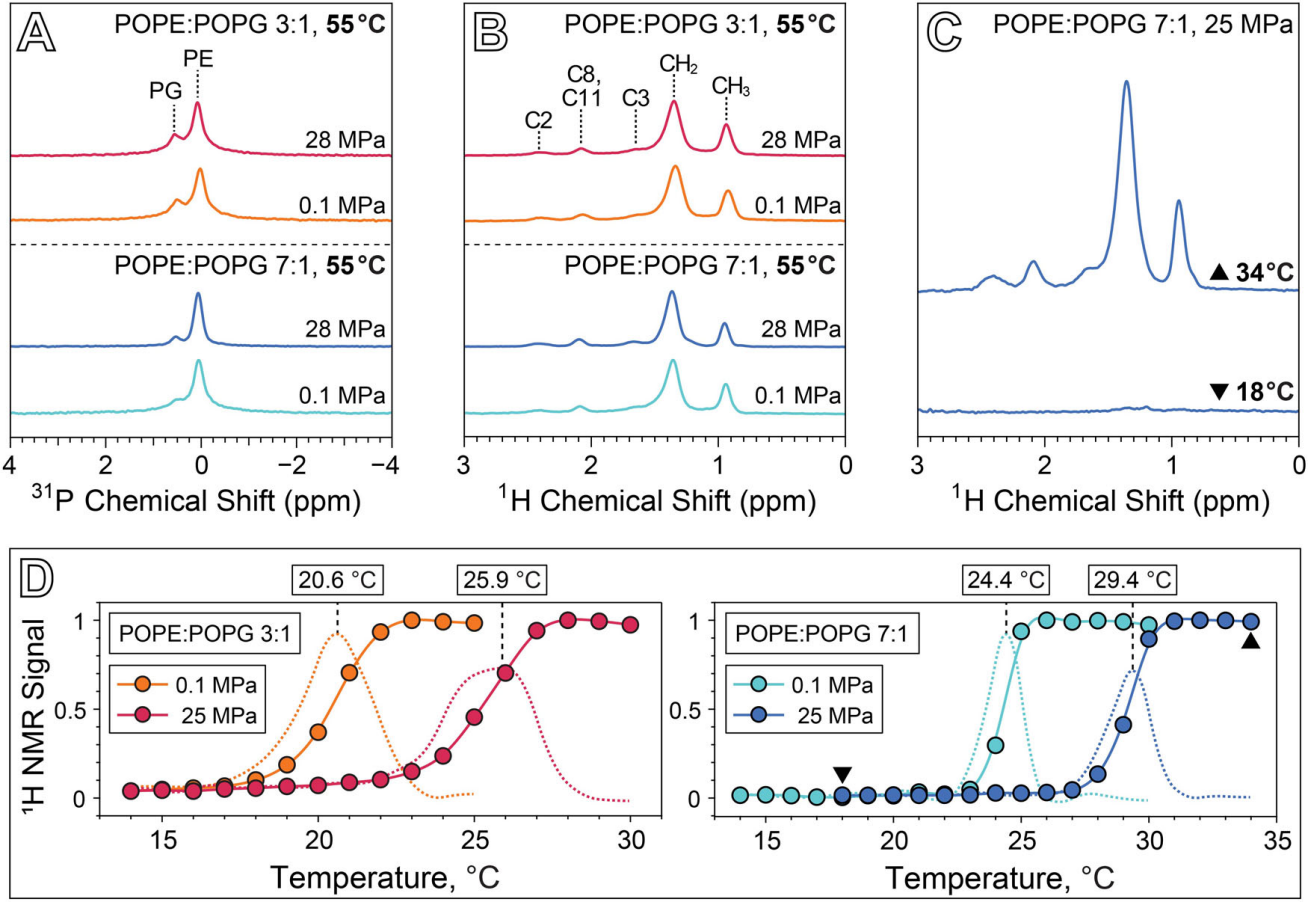
Solid state NMR results measured as a function of temperature and pressure. **A** ^31^P NMR at 4 kHz MAS of 3:1 and 7:1 POPE:POPG at 55 °C and either 0.1 MPa or 28 MPa. **B** ^1^H NMR at 4 kHz MAS of 3:1 and 7:1 POPE:POPG at 55 °C and either 0.1 MPa or 28 MPa, with peak assignments^73^. **C** ^1^H MAS spectra of 7:1 POPE:POPG above (top, 34 °C) and below (bottom, 18 °C) the gel-fluid transition temperature. **D** Plot of ^1^H CH_2_ intensity as a function of temperature for both samples at 0.1 MPa and 25 MPa, normalized to the highest intensity peak in each series. Dashed lines correspond to the derivative of ^1^H signal with temperature. Triangular symbols in the 7:1 POPE:POPG plot correspond to the spectra in **C**.

Solid state ^1^H and ^31^P MAS NMR of 3:1 and 7:1 POPE:POPG vesicles at 55 °C show almost no change in the ^1^H and ^31^P isotropic chemical shifts (*δ*_iso_) or ^31^P T_1_ relaxation time constants between 0.1 MPa and 28 MPa (Fig. 2A, B, Tables 1, 2). In fact, the only perceptible changes in NMR parameters between the two pressure conditions at 55 °C appear to be in the magnitude of *δ*_cs_, the axiality of the ^31^P chemical shift anisotropy (CSA) tensor, and in ^31^P T_2_ relaxation. For both lipid ratios, our measured ^31^P *δ*_cs_ values are consistent with previous measurements^41^, and a slight increase in the ^31^P *δ*_cs_ between 0.1 MPa and 28 MPa is noted for both the PE and PG resonances, which is qualitatively consistent with a reduction in lipid lateral mobility brought on by the application of high pressure. ^31^P T_2_ values increase between 0.1 and 28 MPa for PE and PG at both ratios, with the T_2_ values for the 7:1 POPE:POPG sample in particular increasing by a factor of about 5. The primary effect dominating ^31^P T_2_ relaxation at the magnetic field strength of 9.4 T used in our experiments is the CSA mechanism, where T_2_ depends inversely on the timescale of slow motions such as lateral diffusion and rotational tumbling^42,43^. An increase in ^31^P T_2_ with pressure might thus suggest an increase in the lateral diffusion coefficient, which is counterintuitive given that we generally expect membrane fluidity to decrease with increased pressure. However, CSA-mediated T_2_ relaxation also depends inversely on the square of the order parameter, *S*, meaning the increase in T_2_ with pressure may be unrelated to lateral diffusion, and instead be primarily a result of a reduction in the order parameter in the interphase region of the membrane^42^. Though the application of high pressure is known to increase ordering in the acyl tails^44^, it could also potentially increase the concentration of water in the interphase region and thereby reduce the order parameter of those moieties^45^. Future investigations focusing on measurement of ^13^C-^1^H dipolar couplings to obtain order parameters for the headgroups, glycerol backbone, and acyl tails could aid in supporting this hypothesis^46–49^.

**Table 1:** ^31^P NMR parameters measured for 3:1 POPE:POPG at 55 °C and 0.1 MPa or 28 MPa

3:1 POPE:POPG	0.1 MPa		28 MPa	
	PE	PG	PE	PG
^31^P *δ*_*cs*_ (*δ*_*||*_ - *δ*_iso,_ ppm)	17.4 ppm	19.2 ppm	19.0 ppm	20.0 ppm
^31^P *δ*_iso_ (ppm)	0.02 ppm	0.51 ppm	0.08 ppm	0.56 ppm
^31^P T_1_ (s)	1.35 s	1.35 s	1.34 s	1.31 s
^31^P T_2_ (ms)	3.5 ms	2.2 ms	4.0 ms	3.0 ms

**Table 2:** ^31^P NMR parameters measured for 7:1 POPE:POPG at 55 °C and 0.1 MPa or 28 MPa

7:1 POPE:POPG	0.1 MPa		28 MPa	
	PE	PG	PE	PG
^31^P *δ*_*cs*_ (*δ*_*||*_ - *δ*_iso_)	20.8 ppm	21.9 ppm	22.8 ppm	25.7 ppm
^31^P *δ*_iso_	0.05 ppm	0.49 ppm	0.06 ppm	0.53 ppm
^31^P T_1_	1.39 s	1.29 s	1.35 s	1.27 s
^31^P T_2_	2.8 ms	1.5 ms	11.9 ms	7.9 ms

The gel-fluid transition for both sample compositions under ambient and high pressure conditions was investigated by monitoring the intensity of the acyl tail CH_2_^1^H MAS signal as a function of temperature (Fig. 2C, D). The lack of motional averaging in the gel phase produces ^1^H-^1^H dipolar couplings that exceed the spatial averaging capability of the relatively slow MAS employed in our experiment (4 kHz), resulting in extremely broad peaks for the CH_2_ and other acyl tail moieties. As the membrane transitions into the fluid phase with increasing temperature, motional averaging of dipolar couplings narrows these resonances significantly. This phenomenon is demonstrated in Fig. 2C, which shows the spectra of 7:1 POPE:POPG above and below the transition in a rotor pressurized initially at 25 MPa and 20 °C. Figure 2D plots the CH_2_ intensity as a function of temperature for each membrane composition and pressure condition, where the transition temperature is defined here as the maximum in the derivative of CH_2_ intensity with respect to temperature. ^1^H NMR spectra as a function of temperature for each sample and pressure condition are shown in Supplementary Figs. S1, S2.

Note that the transition temperatures obtained by this method are measured at constant volume. The high pressure sample rotors used in our experiment are sealed with an ideal gas as the pressure-transmitting medium, and thus an increase in temperature must necessarily be coupled with an increase in pressure. This has almost no effect at 0.1 MPa, but as the rotors were initially pressurized at 25 MPa and 20 °C, the pressure varies from 24.4 MPa at 14 °C up to 26.2 MPa at 34 °C. This is also why we have reported the pressure for our NMR measurements taken at 55 °C to be 28 MPa. The gel-fluid transition temperature for 3:1 POPE:POPG increases from 20.6 °C at 0.1 MPa to 25.9 °C at 25.5 MPa, a slope of 0.21 °C/MPa, while the transition temperature for 7:1 POPE:POPG increases from 24.4 °C at 0.1 MPa to 29.4 °C at 25.8 MPa, yielding a slope of 0.19 °C/MPa. The pressure dependence of the transition temperature, or Clapeyron slope, for both membrane compositions is consistent with a value of 0.2 °C/MPa that has been widely observed across a range of phospholipid bilayer systems^3,50,51^. At 0.1 MPa, increasing the POPG content in the membrane from a 7:1 to a 3:1 POPE:POPG ratio lowers the transition temperature by 3.8 °C and broadens the temperature range of the transition. This finding is consistent with previous studies using differential scanning calorimetry, which have demonstrated that the phase transition temperature of PE/PG mixtures varies between the higher transition temperature of PE and the lower transition temperature of PG, in approximate proportion to the lipid concentration ratio^52–54^. Notably, our results show that this effect persists at high pressure, suggesting that to a first approximation, lipid concentration and pressure operate as orthogonal parameters in their influence on membrane dynamics, and that the effect of a particular lipid’s concentration on membrane fluidity may manifest similarly at both low and high pressure conditions.

### Measurement of parameters required for analysis of ^31^P CODEX data

Although the ^31^P CODEX NMR method for measuring lateral diffusion of phospholipids has been described in detail previously^34^, a brief overview of the technique is warranted here for context. The CODEX pulse sequence is applied to a sample undergoing MAS and is divided into three periods. In the first period, rotor-synchronized pulses are applied to recouple the CSA interaction during MAS and impart a phase to each spin that depends on the orientation of its CSA tensor (the spin’s molecular environment) with respect to the magnetic field. This initial phase value is effectively a label for the starting “latitudinal position” of a phospholipid on the surface of a spherical membrane vesicle. In the second period of the pulse sequence, the mixing period, the phase information acquired in the first period is stored and the spin is allowed to sample its environment. In the case of phospholipids on a spherical membrane vesicle, this is when lateral diffusion takes place. The third period of the sequence recouples the CSA once again by rotor-synchronized pulses, and if no lateral diffusion were to occur during the mixing period, the effect of this third period on each spin would be to impart phase equal and opposite to the phase acquired in the first period of the sequence, as the orientation of the CSA tensor will not have changed. In this scenario, the intensity of the resulting NMR signal would be unaffected. However, when lateral diffusion does occur during the second period, the phase value acquired in the third period will not cancel out the spin’s initially encoded phase, resulting in dephasing of the net magnetization and a decrease in NMR signal intensity. The value of *D*_lat_ can then be extracted from the decay of NMR signal as a function of increasing mixing time. As the NMR signal is detected with high resolution under MAS, the signal decay of each ^31^P resonance can be fit to determine *D*_lat_ for all phospholipid species in the membrane in one experiment.

**Fig. 3:**
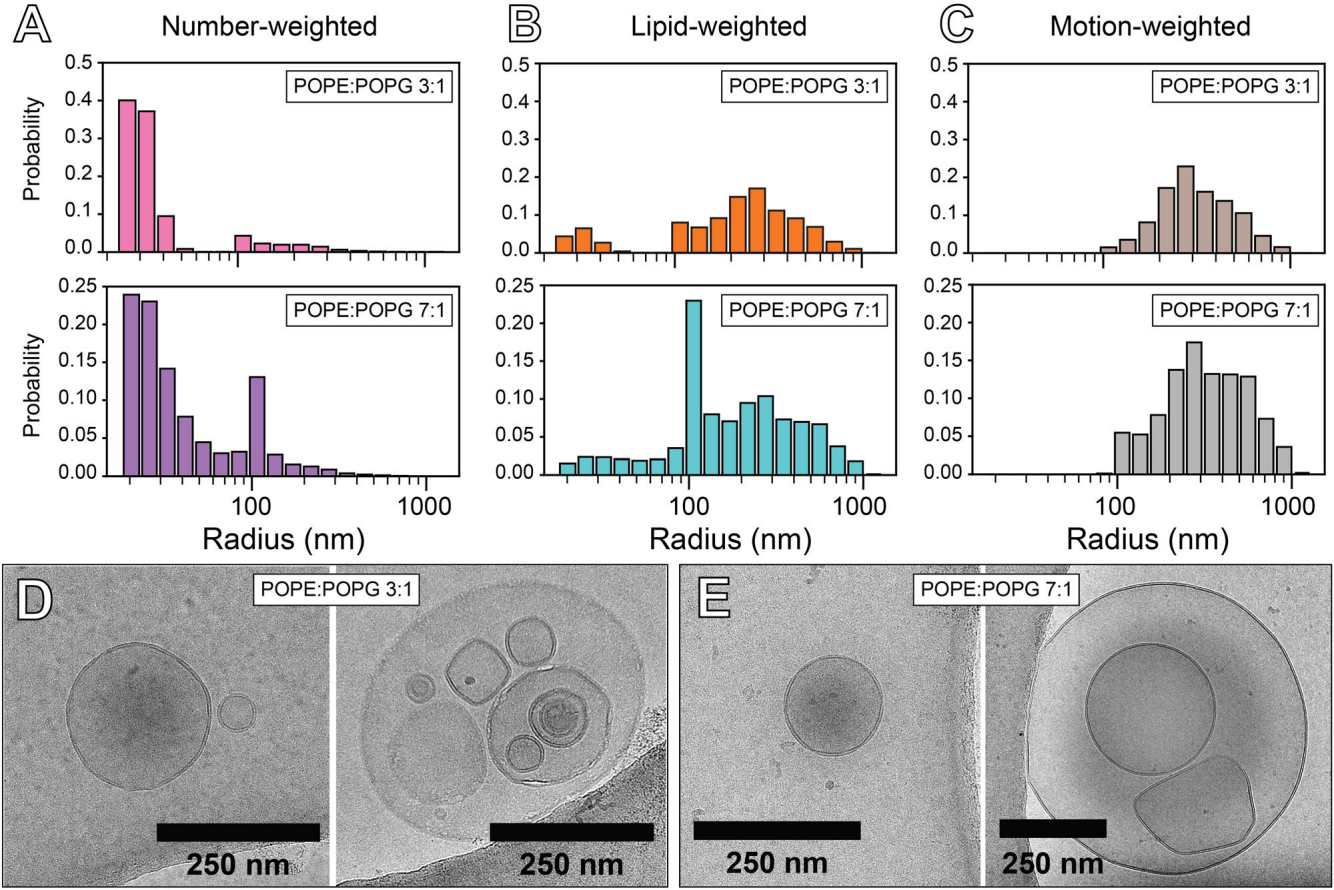
Size distribution and lamellarity of phospholipid samples. **A** Vesicle radius distribution weighted by number of vesicles obtained from DLS. **B** Size distribution from **A** weighted by 8π*r*^2^ accounting for the number of lipids per vesicle. **C** Size distribution from **B** weighted by contribution to the observed ^31^P CODEX signal. **D,E** CryoEM images of the 3:1 (**D**) and 7:1 (**E**) POPE:POPG samples, each showing a unilamellar vesicle with radius <100 nm (left), and a representative image of a larger vesicle (>100 nm) with smaller vesicles contained within it (right).

In the case of phospholipid vesicles, the process to fit the CODEX decay curve involves simulation of the expected magnetization as a function of mixing time by modeling the dephasing effect of the CSA recoupling periods on a population of spins undergoing gaussian diffusion on a spherical surface of radius *r*^34,55^. We have written an open-source, Python-based program with a graphical interface to perform this simulation and extract *D*_lat_ from ^31^P CODEX decay data (Supplementary Information). Two experimentally derived sample-dependent parameters are required as inputs for this simulation: the ^31^P CSA for each phospholipid species, and the distribution of phospholipid vesicle radii in the sample. ^31^P CSA values for POPE and POPG in each sample were obtained by simulation and fitting of the spinning sideband manifold in ^31^P NMR spectra collected at 2 kHz MAS (Supplementary Fig. S3)^56^. Simulation of the magnetization decay curve accounts for the distribution of vesicle sizes by summing over the calculated expected magnetization contribution for each vesicle radius in the distribution, weighted by the probability of finding a lipid in a vesicle at each radius (Fig. 3). The weighting function for a given *r* is given by the number-weighted probability of finding a vesicle with radius *r*, *P*(*r*), obtained by dynamic light scattering (DLS), multiplied by twice the surface area of the vesicle, 8π*r*^2^, to account for the number of ^31^P nuclei in both the outer and inner leaflets of the membrane. Finally, overall vesicle tumbling combined with lipid lateral diffusion during the CSA recoupling period weights signal contributions by exp(-*τ*_echo_/*τ*_c_), with *τ*_echo_ being the duration of the recoupling period and *τ*_c_ being the rotational correlation time, given in ref. ^34^

1 $$\frac{1}{{\tau }_{{{\rm{c}}}}}=\frac{3{k}_{{{\rm{B}}}}T}{4\pi \eta {r}^{3}}+\frac{6{D}_{{{\rm{lat}}}}}{{r}^{2}}$$

where *η* is the viscosity of the solution, for which we have used a value of 5.5 * 10^−4^ Pa*s for our samples at 55 °C based on previous measurements of the viscosity of aqueous NaCl solutions at elevated pressure and temperature^57^. The weighting effect of rotational motion during the recoupling period for the case of *D*_lat_ = 10^−12^ m^2^s^−1^ is shown in Fig. 3C, indicating that vesicles with radii below about 100 nm do not contribute to the observed ^31^P CODEX signal.

Vesicle size distributions obtained by DLS only account for the size of the outermost vesicle and cannot measure vesicles contained within their interior. As ^31^P NMR is sensitive to all vesicles and not just the outermost ones, the sample must be prepared as unilamellar vesicles for size distributions measured by DLS to reflect the true size distribution of the sample. However, typical preparation methods for producing unilamellar vesicles have been observed to still yield a population of multilamellar vesicles (MLVs)^58^. Measurement of vesicle lamellarity can be performed by fluorescence or radical quenching methods^59^, but these techniques rely on the incorporation of tagged lipids within the membrane, potentially affecting the lateral diffusion of other phospholipids. To assess the lamellarity of our samples without incorporating molecular tags, we performed cryo-electron microscopy (cryoEM, Fig. 3D, E). We observed unilamellar vesicles with radii below 100 nm, either isolated by themselves or contained within the interior of larger, >100 nm radius vesicles. Many of these larger vesicles, despite technically being MLV’s, lack the characteristic “onion” morphology typical of MLVs, and thus their membrane properties are likely more similar to unilamellar vesicles than true MLVs. Based on these observations, we can assume that the majority of phospholipids within the interior of the large vesicles belong to unilamellar vesicles with radii ≤100 nm, which do not contribute to the observed ^31^P CODEX signal due to the weighting effect of rapid rotational motion during the recoupling period. With this assumption, the vesicle size distribution obtained by DLS closely approximates the true vesicle size distribution relevant for the CODEX decay.

In our analysis, we have also assumed that the size distributions of these samples remain unchanged throughout the process of sample preparation and data collection. Verification of the size distribution of the vesicles post-MAS and post-pressurization is complicated by rapid depressurization of the rotor upon loosening of the retaining screw, which will affect vesicle morphology and make measurements of the size distribution after the NMR experiment highly inaccurate. However, the buffer used in these samples is relatively salty (50 mM NaCl), which itself plays a significant role in preventing aggregation of vesicles during the experiment. The centrifugal effect of MAS in a 5 mm rotor (3.3 mm inner diameter) at 4 kHz produces a relative centrifugal force of 212,500 x *g* at the inner wall of the rotor, which is about the centrifugal force experienced by a liposome pellet under ultracentrifugation. From our experience, resuspension of ultracentrifuged liposome pellets into buffer by vortexing yields a vesicle size distribution matching that of the solution prior to ultracentrifugation. With respect to the effect of pressurization in the high pressure MAS rotor, the process proceeds slowly, and without a discontinuous jump in pressure that might shear or stress the vesicles. Therefore, we expect no adverse effects on size distribution resulting from the increase in pressure. Nevertheless, while our observations suggest that vesicle size distributions are likely unaffected by the combined effects of MAS and pressurization, a more comprehensive understanding of these influences on membrane systems will require further targeted investigation.

### Effect of pressure and membrane composition on phospholipid lateral diffusion

**Fig. 4:**
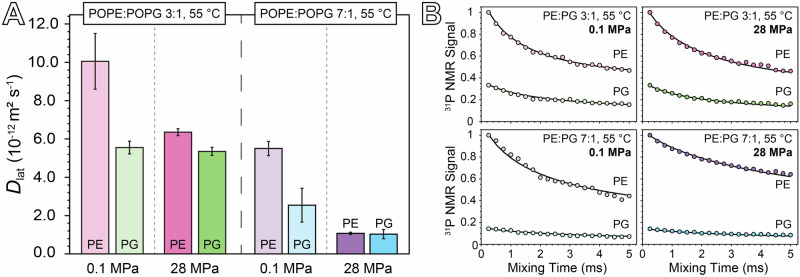
Phospholipid lateral diffusion coefficients obtained by ^31^P CODEX NMR. **A** Comparison of *D*_lat_ values measured for POPE and POPG at 0.1 MPa and 28 MPa for both the 3:1 and 7:1 samples. Error bars reflect a 95% confidence interval based on the results of the CODEX decay curve fitting process. **B** ^31^P CODEX signal for PE and PG normalized to PE as a function of mixing time in the CODEX sequence for each ^31^P resonance, plotted with the CODEX decay curve obtained by simulation to obtain *D*_lat_.

Figure 4 and Table 3 compare the lateral diffusion coefficients (*D*_lat_) obtained by fitting the ^31^P CODEX decay curves obtained for each sample at 55 °C under pressures of 0.1 MPa and 28 MPa. Spectra associated with the ^31^P NMR signal values shown in Fig. 4B are provided in Supplementary Fig. S4, and the program used for simulating the CODEX decays is provided in Supplementary Folder 1. The *D*_lat_ values for both POPE and POPG in our samples are consistent with previously reported experimental and theoretical data for phospholipids near room temperature^33,40,44,60–63^. Notably, at 0.1 MPa, the *D*_lat_ values for both POPE and POPG in the 3:1 POPE:POPG sample are approximately twice those observed in the 7:1 POPE:POPG sample. This difference becomes more pronounced at 28 MPa, where the *D*_lat_ of both POPE and POPG in the 3:1 sample exceed those in the 7:1 sample by more than fivefold. Additionally, lateral diffusion in the 7:1 membrane is overall more sensitive to pressure than in the 3:1 sample. Specifically, the *D*_lat_ of POPE decreases by a factor of five in the 7:1 composition and by 1.5 in the 3:1 composition when the pressure is increased from 0.1 MPa to 28 MPa. In contrast, the *D*_lat_ of POPG decreases by a factor 2.5 in the 7:1 sample but is nearly unaffected by pressure in the 3:1 sample. These results demonstrate that membrane composition significantly influences not only the absolute values of *D*_lat_ under pressure, but also the magnitude of its change in response to pressure.

**Table 3:** *D*_lat_ values in units of 10^−12^ m^2^s^−1^ for the two membrane compositions measured at 55 °C and 0.1 MPa or 28 MPa

*D*_lat_ (10^−12^ m^2^s^−1^)	0.1 MPa		28 MPa	
	POPE	POPG	POPE	POPG
POPE:POPG 3:1	10.04 ± 1.45	5.55 ± 0.33	6.35 ± 0.18	5.35 ± 0.21
POPE:POPG 7:1	5.50 ± 0.37	2.55 ± 0.88	1.08 ± 0.06	1.04 ± 0.24

Error is represented as a 95% confidence interval based on the results of the CODEX decay curve fitting process.

To better understand how membrane composition modulates the pressure-dependent behavior of phospholipid lateral diffusion, it is essential to consider the distinct physical properties of POPE and POPG. The headgroups of PE and PG differ significantly in their electrostatic charges and hydrogen-bonding capacities. PE is zwitterionic, with a net charge of zero, arising from a positively charged ammonium group and a negatively charged phosphate group. In contrast, PG carries a net charge of −1, due to the only charged moiety being the negatively charged phosphate group. Furthermore, their hydrogen-bonding characteristics differ: PE, with its ammonium group as a strong donor and its phosphate as a strong acceptor, is more capable of participating in hydrogen bonding networks, while PG primarily serves as a hydrogen bond acceptor through its phosphate group, albeit with some contribution from the glycerol group^52^.

Previous simulations of a 3:1 POPE:POPG membrane have demonstrated that POPE and POPG engage in hydrogen bonding, either directly or through water bridges, with POPE displaying a greater affinity for POPG than for other POPE molecules, and POPG interacting nearly exclusively with POPE^40^. The propensity for PG to act as a hydrogen bond acceptor disrupts the hydrogen bonding network between PE groups, and this effect is likely what accounts for the increase in *D*_lat_ observed for both POPE and POPG at 0.1 MPa when the concentration of POPG is increased. While the phenomenon of hydrogen bond network disruption by POPG is likely still present at 28 MPa, the larger change in *D*_lat_ with pressure for POPE compared to POPG suggests that additional pressure-dependent mechanisms may be playing a role.

A key distinction between POPE and POPG is their influence on membrane curvature, which is quantified by the monolayer spontaneous curvature parameter for a lipid species, *c*_0_^64^. This parameter represents the radius of an unstressed monolayer comprised solely of that lipid and is given in units of inverse length. The spontaneous curvature is correlated with the volumetric shape of the lipid: a *c*_0_ value of 0 corresponds to a cylindrical shape, while a non-zero value indicates a conical shape. A positive *c*_0_ corresponds to a cone narrowing toward the lipid tails, whereas a negative *c*_0_ indicates a cone widening toward the tails. As *c*_0_ is directly related to the volume of a lipid, it can be highly dependent on pressure. The values of *c*_0_ for both POPE and POPG at ambient pressure and 35 °C have previously been measured to be −0.35 Å^−1^ and 0.02 Å^−1^ respectively^65^, indicating that POPE has a tail-dominated conical shape, while POPG is approximately cylindrical. Pressure has been shown to increase the value of *c*_0_ for negative-curvature lipids due to ordering of the hydrocarbon tails^25^, which reduces the tail region’s volume more than the headgroup region’s volume. This tail-volume restriction at high pressure may explain the reduction in *D*_lat_ observed at 28 MPa for both POPE and POPG in both the 3:1 and 7:1 samples. Additionally, if the pressure dependence of *c*_0_ were less pronounced for POPG than for POPE, this could also explain why the *D*_lat_ of POPE is more affected by pressure than that of POPG across both membrane compositions. However, support for this explanation requires additional studies investigating the influence of pressure on spontaneous curvature.

The difference in pressure sensitivity of *D*_lat_ between the two phospholipid ratios may be a result of composition-dependent inter-lipid interactions. At 28 MPa, the *D*_lat_ value for POPE roughly matches that of POPG in both the 3:1 and 7:1 samples, suggesting that at high pressure, the lateral diffusion of POPG seems to act as a lower limit for lateral diffusion in the membrane overall, possibly due to interactions between POPE and POPG. Furthermore, doubling the concentration of POPG from 7:1 to 3:1 significantly reduces the pressure-induced change in *D*_lat_ for POPE, decreasing the factor of reduction from 5 to 1.5. This suggests that POPG exerts a fluidizing effect on the membrane that keeps the *D*_lat_ of POPE from dropping below the *D*_lat_ of POPG, even at high pressures.

Recent molecular dynamics simulations suggest that the spontaneous curvature of lipid dimers may not follow a simple additive model. Instead, interactions such as hydrogen bonding can give rise to a phenomenon called diffusional softening—a proposed mechanism where curvature-based lipid sorting interacts with membrane undulations to influence bilayer mechanics^66^. In the context of our results, the presence of hydrogen-bonded PE-PG dimers, which exhibit less pronounced negative curvature relative to PE-PE dimers, introduces heterogeneity into the local curvature landscape of the membrane. Such heterogeneity may enable membrane undulations to more readily reorganize lipids, minimizing the energy of the membrane and mitigating the stiffening effects of pressure. Although these concepts are drawn from simulations and are not yet experimentally verified in this system, the apparent convergence of *D*_lat_ values for POPE and POPG at high pressure in both lipid compositions is consistent with the notion that curvature sorting of PE-PG dimers could contribute to the fluidizing effect of PG. In essence, by forming such dimers, the lateral diffusion of PE becomes increasingly coupled to that of PG, reducing the disparity in their mobilities and enhancing overall membrane fluidity under high-pressure conditions. Moreover, in the 3:1 sample, the increased fraction of PE-PG dimers compared to the 7:1 sample may enhance the influence of diffusional softening and thus reduce the pressure sensitivity of *D*_lat_. Confirming this interpretation, however, will require further experimental and theoretical investigations to fully elucidate how inter-lipid interactions, curvature sorting, and pressure collectively shape membrane fluidity.

## Conclusion

In this study, we employed in situ, high pressure MAS NMR to investigate the lateral diffusion (*D*_lat_) of phospholipids in model bacterial membranes by ^31^P CODEX NMR, demonstrating how membrane composition can significantly influence membrane fluidity under high pressure conditions. We found that in mixed-lipid membranes comprised of POPE and POPG, the concentration of POPG plays a crucial role in preserving membrane fluidity, particularly by moderating the impact of pressure on the lateral diffusion of POPE. These results suggest that interactions between PE and PG are capable of regulating membrane dynamics in response to pressure. Additionally, our manual pressurization strategy and open-source Python program for extracting *D*_lat_ from CODEX decay curves together offer an accessible means to embark on future high pressure solid state NMR studies to investigate these lipid interactions in more detail.

Despite the simplicity of our binary lipid model compared to real bacterial membranes, this study provides a clear demonstration of the principle of homeoviscous adaptation to pressure. Consider a hypothetical microorganism that thrives at 0.1 MPa and 55 °C with a membrane composed of 7:1 POPE:POPG. If this organism were exposed to a 28 MPa environment without adjusting its lipid composition, the lateral diffusion of POPE—the majority phospholipid in its membrane—would decrease by a factor of 5, potentially hindering biological functions. However, by increasing the concentration of POPG in its membrane, the organism could restore the lateral diffusion of both POPE and POPG at 28 MPa, maintaining a fluidity comparable to that at 0.1 MPa.

The complexity of lipidome changes observed in real biological membranes in response to pressure—encompassing alterations in phospholipid headgroups, tail lengths, degrees of unsaturation, branching, and other chemical modifications—points to the multifunctional nature of membrane composition and the coordinated intricacy of the homeoviscous response. While there may be multiple strategies that can maintain membrane fluidity at high pressure, such as adjusting headgroup concentrations or altering tail structure, these changes must be carefully balanced against other membrane functions. For example, while increasing the proportion of PG relative to PE enhances membrane fluidity at high pressure, it also alters the surface charge of the membrane, potentially affecting other critical biological processes. Unraveling the precise rationale behind an organism’s specific lipid adaptations to environmental stimuli such as pressure requires a holistic understanding of the membrane and its interactions with its surroundings. Further studies investigating the structure-property relationships of lipids will be essential to advancing our understanding of these complex mechanisms.

### Experimental Details

#### Preparation of phospholipid liposome samples

Phospholipids used for liposome preparation, POPE, 1-palmitoyl-2-oleoyl-sn-glycero-3-phosphoethanolamine (16:0-18:1 PE), and POPG, 1-palmitoyl-2-oleoyl-sn-glycero-3-phosphoglycerol (16:0–18:1 PG), were obtained from Avanti Polar Lipids (Alabaster, AL, United States) as 25 mg/mL chloroform solutions and used directly without further purification. All other chemicals used in this work were obtained from Sigma-Aldrich (St. Louis, MO, United States) and used without further purification.

The following protocol was used to prepare mixed lipid liposome samples at either a 3:1 or 7:1 POPE:POPG molar ratio. A chloroform solution containing a total dry lipid mass of 35 mg of POPE and POPG at the specified molar ratio was dried over argon in a 10 mL glass round bottom flask to form a thin film. The lipid film was then exposed to vacuum overnight to remove residual chloroform. The film was hydrated by vortexing with 8 mL 0.22 μm-filtered MES buffer (50 mM MES, 50 mM NaCl, 2 mM EDTA, pH 5.5), then bath sonicated for 30 min. The solution was subsequently frozen in liquid nitrogen and then thawed in a 45 °C bath for three cycles. The solution was then divided into four 2 mL aliquots and ultracentrifuged at 22,000 rpm in a Beckman Coulter Optima MAX-TL ultracentrifuge (Beckman-Coulter, Inc., Brea, CA, United States) for 1 h to yield four liposome pellets. After decanting the supernatant, two of these pellets were then transferred into a 5 mm high pressure rotor for solid state NMR experiments using a previously reported 3D-printed centrifugal device^67^ placed in an Eppendorf 5810 R swinging-bucket centrifuge (Eppendorf, Hamburg, Germany) and spun at 3197x*g* for 5 min.

#### Liposome size distribution measurements by dynamic light scattering (DLS)

A Wyatt Technology DynaPro Plate Reader (Wyatt Technology, Santa Barbara, CA, United States) was used for DLS measurements to obtain liposome size distributions. For each sample, one of the ultracentrifuged pellets was resuspended in 1 mL of MES buffer, and then subsequently diluted by a factor of 1/100 for DLS analysis. Data was acquired at 25 °C with 5 scans per well. The resulting intensity autocorrelation data was then fit using regularization analysis in the DYNAMICS7 software to obtain the particle number-weighted size distribution for each liposome sample.

#### Liposome lamellarity measurements by cryo-electron microscopy (cryoEM)

Samples for cryoEM measurements were prepared from the resuspended liposome pellet solution that was used for DLS. Samples were vitrified using a Leica EM-GP plunge freezer (Leica Microsystems GmbH, Wetzlar, Germany) and imaged using a 200 kV FEI Talos Arctica cryoelectron microscope (FEI Company, Hillsboro, OR, United States). Images were processed with high-pass filtering to increase visual sharpness using ImageJ software^68^.

#### Preparation of sample rotors for ambient-pressure and high pressure MAS NMR experiments

Two 5 mm high pressure MAS rotors (Phoenix NMR LLC, Loveland, CO, United States) were prepared for NMR experiments. One rotor was filled with 96.8 mg of 7:1 POPE:POPG liposomes, while the other was filled with 100.4 mg of 3:1 POPE:POPG liposomes. After performing NMR experiments on these two samples at ambient pressure (0.1 MPa), each sample was pressurized using the experimental apparatus shown in Fig. 1. The rotor was secured in a small plastic collet and placed standing upright into a 50 mL stainless-steel high pressure vessel (Parr Instrument Company, Moline, IL, United States), rated to contain a maximum pressure of 34.5 MPa. To pressurize the vessel, and thereby pressurize the contents of the rotor, the vessel was first evacuated to remove air from the headspace, then pressurized to 25 MPa with helium gas by manual pumping using a Hill MK5 hand pump (Ernest H. Hill Ltd., Sheffield, United Kingdom) with the gas inlet modified using a 3D-printed adapter to accept the low-pressure regulated output from a helium gas cylinder. Once the desired pressure was reached, the system was allowed to equilibrate at 20 °C for 10 min, and then the vessel was depressurized at a rate of about 5 MPa per minute. The rotor was then removed from the vessel, and successful pressurization of the rotor contents was verified by measuring a helium mass gain of 2.3 mg for the 7:1 POPE:POPG rotor and 2.0 mg for the 3:1 POPE:POPG rotor. Pressurized rotors were transported to the NMR spectrometer in a secondary containment vessel made of Delrin. Care was taken during handling of the pressurized rotors to wear appropriate personal protective equipment, and to always direct the capped end of the rotor away from the operator and other individuals in the laboratory, as the cap may act as a projectile if it were to fail. After performing NMR experiments, the mass of each rotor was measured and found to remain unchanged to within ±0.2 mg. Removal of the retaining screw from each rotor was accompanied by an audible hiss due to depressurization of the contents.

#### Solid state NMR instrumentation and experiments

NMR experiments were performed at 9.4 T magnetic field using a Bruker Avance III 3-channel spectrometer (Bruker Corp., Billerica, MA, United States) operating at 400.2 MHz for ^1^H, 162.0 MHz for ^31^P, and 100.2 MHz for ^79^Br. A Varian T3 triple-resonance 5 mm MAS probe in HPC tuning mode with a Varian MAS controller (Varian, Palo Alto, CA, United States) was used for all NMR experiments. Sample temperature control was achieved by flowing the low-temperature output of an SP FTS AirJet XR (Scientific Products, Warminster, PA, United States) through a Varian solids upper heating stack, which was controlled by an L950 temperature controller (Highland Technology, Inc., San Francisco, CA, United States). Serial data communication to the temperature controller was handled by an MS-DOS calibration interface program running in a DOSBox 0.74-3 emulation environment. Temperature calibration of the system over a range of 10 - 70 °C was performed using dry ethylene glycol (dried over 3 Å molecular sieves) in a high pressure rotor^69,70^. The sample temperature was calibrated over this range at both 2 kHz and 4 kHz MAS to account for the difference in frictional heating between the two spinning rates. The stator was set to the magic angle by optimization of rotor echo intensity in the time-domain ^79^Br signal of KBr^71^. ^1^H chemical shifts were externally referenced to the trimethylsilyl resonance of sodium trimethylsilylpropanesulfonate (DSS) in D_2_O at 0 ppm, and ^31^P chemical shifts were externally referenced to 85% phosphoric acid at 0 ppm.

^1^H NMR spectra were collected at 4 kHz MAS as a pulse-acquire experiment with presaturation for water suppression, using a π/2-pulse of 5.0 µs and a recycle delay of 1.0 s. Variable temperature ^1^H NMR data were collected with 10 min of equilibration time at each temperature point prior to acquisition. Acquisition of ^31^P NMR spectra and measurement of ^31^P T_2_ values were performed at 4 kHz MAS using the Hahn echo pulse sequence with rotor period integer multiple echo delays and a π/2-pulse of 6.0 µs and a recycle delay of 1.5 s. ^31^P T_1_ values were measured at 4 kHz MAS using the inversion recovery pulse sequence with a recycle delay of 10.0 s. Slow-spinning ^31^P spectra for determination of the ^31^P chemical shift anisotropy were collected at 2 kHz MAS and 55 °C, with a recycle delay of 10 s. ^31^P centerband-only detection of exchange (CODEX) experiments^31,34^ were performed at 4 kHz MAS (with spin rate stability < ±1 Hz) using direct excitation of ^31^P, three π-pulses during the rotor-synchronized dephasing and rephasing pulse periods, mixing times at integer multiples of the rotor period, a z-filter of one rotor period prior to acquisition, 2048 scans at each mixing time, and a recycle delay of 1.5 s.

#### Data processing and analysis

All NMR spectra were processed in Bruker TopSpin 4.3.0 and Fourier transformed using the sine-squared window function with a sine bell shift of 2. ^31^P T_1_ and T_2_ values were determined using the relaxation data fitting suite in TopSpin. Fitting of ^31^P signal area and determination of *δ*_cs_ from slow-spinning ^31^P MAS spectra were performed using DMFit^72^. Plots of NMR signal intensity vs. temperature for each sample were made by measuring the ^1^H intensity at 1.35 ppm (corresponding to the lipid tail CH_2_ groups) for each temperature. The gel-fluid phase transition temperature for each sample was determined in MATLAB by first generating a curve via cubic smoothing spline interpolation of the experimental data with a smoothing parameter of 0.975, and then taking the numerical derivative of this curve with respect to temperature. The phase transition temperature was then taken as the temperature corresponding to the peak maximum in the derivative plot.

Extraction of the phospholipid lateral diffusion coefficients, *D*_lat_, from the ^31^P CODEX decay curves was performed based on the method introduced by Macdonald and coworkers^34^. Fitting of the CODEX decay curves was performed using a homebuilt Python program with a graphical user interface (see Code Availability for more information). A detailed description of the program may be found in the associated readme file.

#### Use of large language models for manuscript and code preparation

During the preparation of this work, the authors used ChatGPT-4o (OpenAI, San Francisco, United States) as a polishing and editing tool to suggest alternative phrasing for some author-written sentences and improve the style and flow of the manuscript. After using this service, the authors reviewed and edited the content as needed and take full responsibility for the content of the publication. Additionally, ChatGPT-4o was used to improve the readability, organization, and commenting of the Python code written by the authors. The authors are solely responsible for the underlying architecture, data analysis methodology, and decisions made relating to the code implementation.

## Supplementary information

Supplementary Information

Description of Additional Supplementary Files

Supplementary Data 1

## Data Availability

The source data that support the findings of this study are available from the corresponding author upon reasonable request.

## Code availability

The Python program used in this work to fit ^31^P CODEX decay data is available as part of the Supplementary Data 1, and it may also be downloaded from a GitHub repository at https://github.com/2top/. The CODEX NMR peak integrals and liposome size distribution data used to obtain the lateral diffusion coefficients in this work are included along with the program in Supplementary Data 1 as well.
