# Supplementary material for: Measurement of phospholipid lateral diffusion at high pressure by in situ magic-angle spinning NMR spectroscopy
Source: Commun Chem. 2025 Feb 14;8:49. doi: 10.1038/s42004-025-01449-7 (PMC11828890; doi:10.1038/s42004-025-01449-7)
Supplement: Supplementary file 1 — Supplementary Information [file 42004_2025_1449_MOESM1_ESM.pdf]

Supplementary Information

Measurement of phospholipid lateral diffusion at high pressure by *in situ* magic-angle spinning NMR spectroscopy

Thomas M. Osborn Popp,[1,2,‡,*] Mithun Karthikeyan,[2] Elias M. Herman,[2] Andrew C. Dufur,[2] Costantino Vetriani,[3,4] Andrew J. Nieuwkoop[1,*]

1. Department of Chemistry and Chemical Biology, Rutgers University, Piscataway, New Jersey 08854, USA
2. Department of Chemistry, Oregon State University, Corvallis, OR 97331, USA
3. Department of Marine and Coastal Sciences, Rutgers University, New Brunswick, NJ 08901, USA
4. Department of Biochemistry and Microbiology, Rutgers University, New Brunswick, NJ 08901, USA

‡ Current address: Department of Chemistry, Oregon State University, Corvallis, OR 97331, USA

*Correspondence to: osbornpt@oregonstate.edu, an567@rutgers.edu

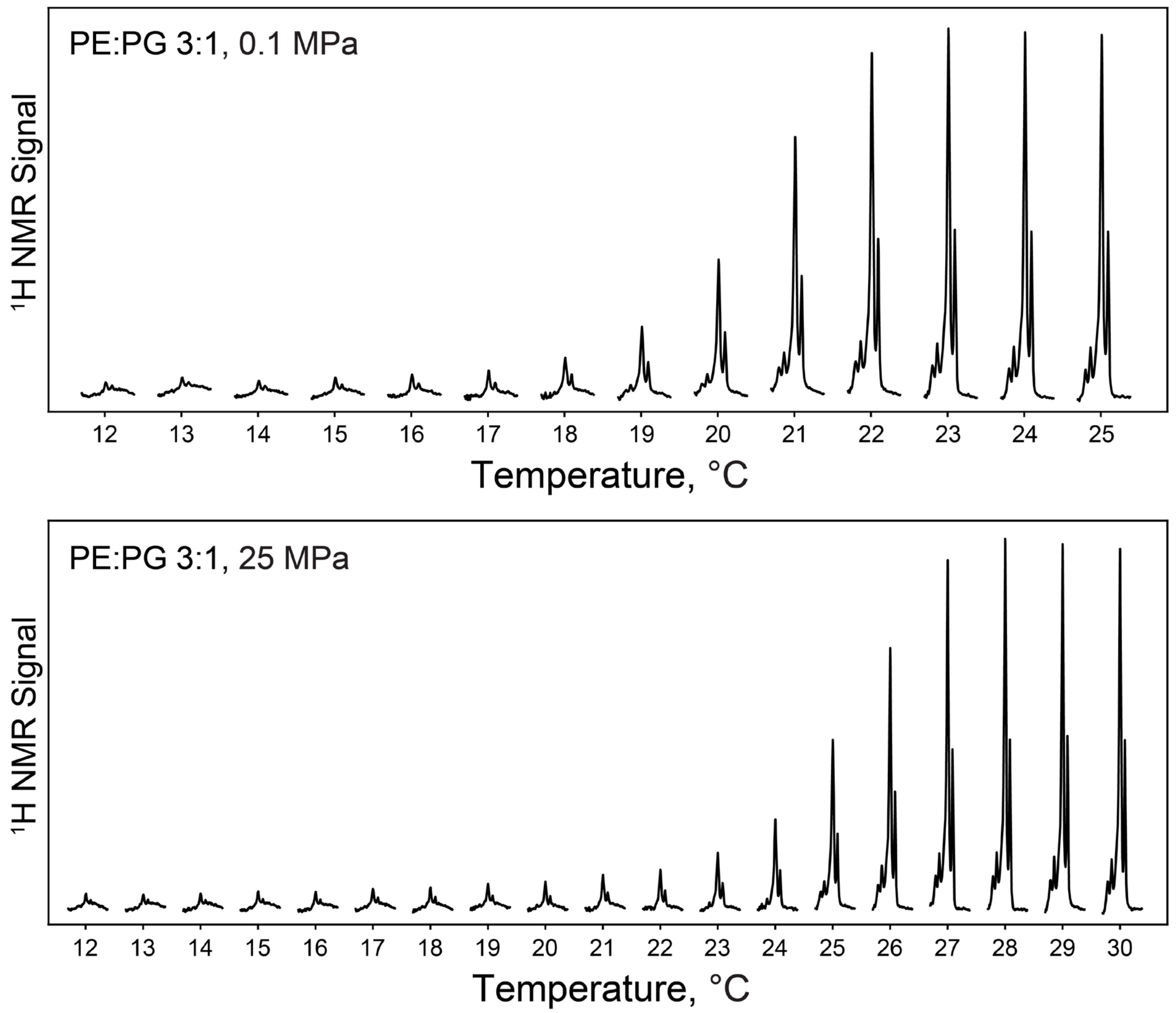

Supplementary Figure S1. Experimental ^{1}H 1D spectra spanning between 0 and 3 ppm as a function of temperature for 3:1 PE:PG at both 0.1 MPa and 25 MPa.

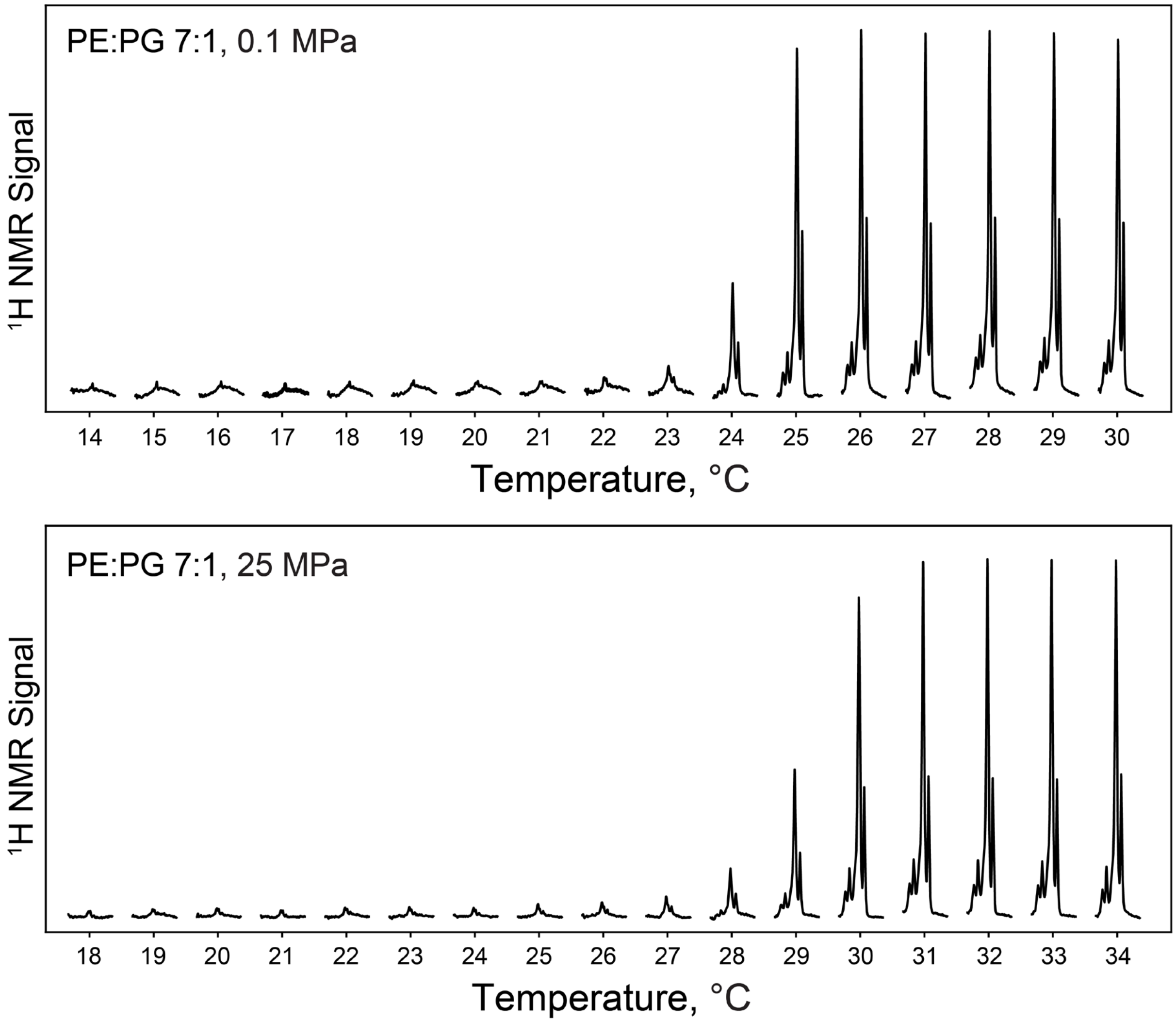

Supplementary Figure S2. Experimental ^{1}H 1D spectra spanning between 0 and 3 ppm as a function of temperature for 7:1 PE:PG at both 0.1 MPa and 25 MPa.

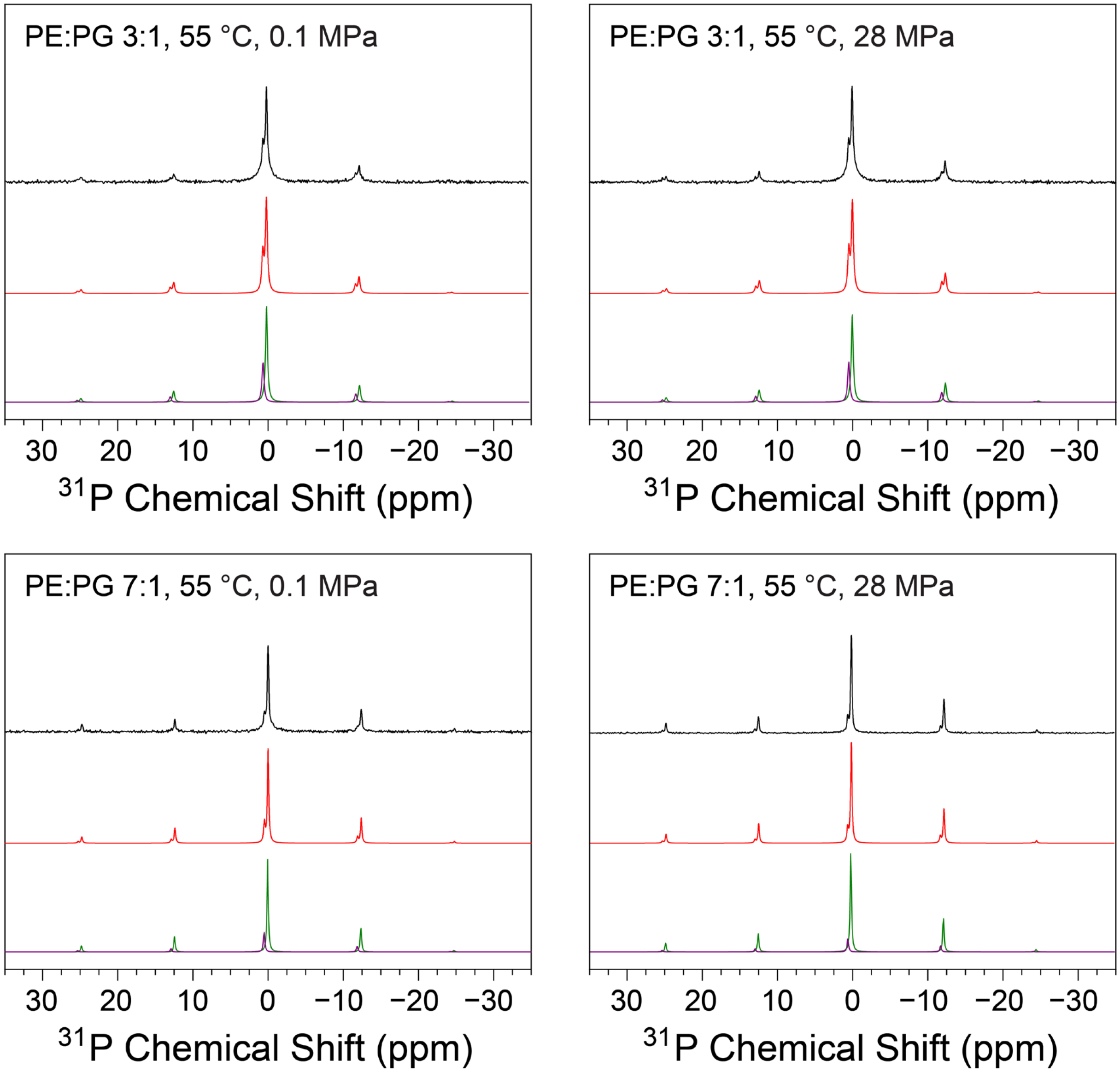

Supplementary Figure S3. Experimental ^{31}P 1D spectra taken at 2 kHz MAS for each sample and condition (black), with simulated spectra (sum: red, PE: green, PG, purple) used to extract δ_{CS}.

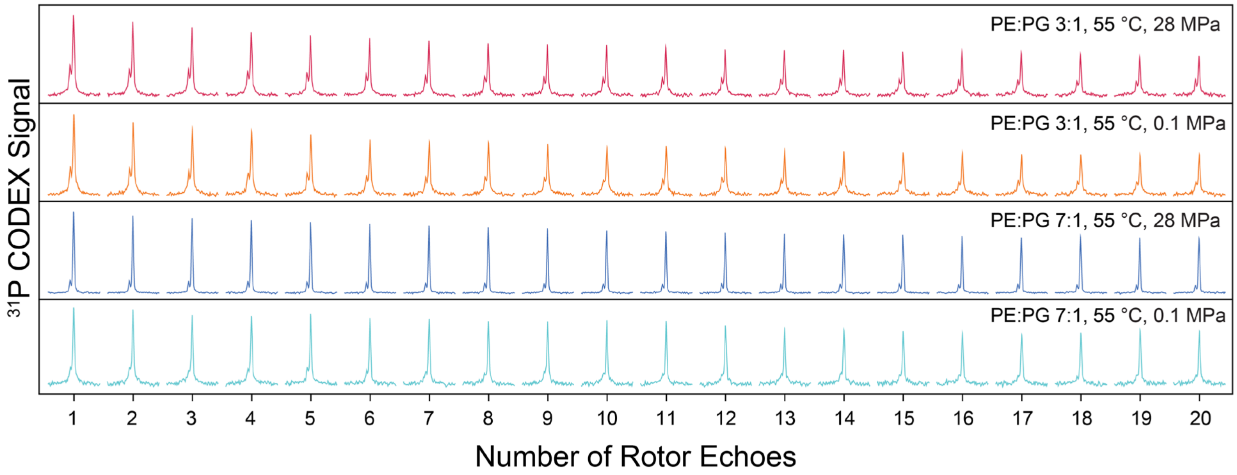

Supplementary Figure S4. Experimental ^{31}P 1D spectra from each CODEX experiment spanning between +3 ppm and -3 ppm as a function of the number of rotor echoes used in the mixing period of the pulse sequence.
