# Supplementary material for: Measurement of phospholipid lateral diffusion at high pressure by in situ magic-angle spinning NMR spectroscopy
Source: Commun Chem. 2025 Feb 14;8:49. doi: 10.1038/s42004-025-01449-7 (PMC11828890; doi:10.1038/s42004-025-01449-7)
Supplement: Supplementary file 2 — Description of Additional Supplementary Files [file 42004_2025_1449_MOESM2_ESM.pdf]

# Description of Additional Supplementary Files

**File name: Supplementary Data 1**

**Description:** This supplementary folder contains a Python-based graphical user interface program composed of 3 files, main.py, states.py, and functions.py, along with a readme file and a list of package requirements. Additionally, it also includes folders containing the experimental 31P CODEX decay data and liposome size distributions that can be used as inputs to the Python GUI to obtain the phospholipid lateral diffusion coefficients.
